# Meta-analysis of the quantitative assessment of lower extremity motor function in elderly individuals based on objective detection

**DOI:** 10.1186/s12984-024-01409-7

**Published:** 2024-06-26

**Authors:** Wen Liu, Jinzhu Bai

**Affiliations:** 1 Rehabilitation Medicine Center, The Second Affiliated Hospital and Yuying Children’s Hospital, Wenzhou Medical University, Wenzhou, China; 2 Department of Spine and Spinal Cord Surgery, Beijing Boai Hospital, China Rehabilitation Research Centre, Beijing, China; 3 School of Rehabilitation Medicine, Capital Medical University, Beijing, China

**Keywords:** Meta-analysis, Elderly individuals, Lower extremities, Motor function, Rehabilitation assessment, Detection, Deep learning

## Abstract

**Objective:**

To avoid deviation caused by the traditional scale method, the present study explored the accuracy, advantages, and disadvantages of different objective detection methods in evaluating lower extremity motor function in elderly individuals.

**Methods:**

Studies on lower extremity motor function assessment in elderly individuals published in the PubMed, Web of Science, Cochrane Library and EMBASE databases in the past five years were searched. The methodological quality of the included trials was assessed using RevMan 5.4.1 and Stata, followed by statistical analyses.

**Results:**

In total, 19 randomized controlled trials with a total of 2626 participants, were included. The results of the meta-analysis showed that inertial measurement units (IMUs), motion sensors, 3D motion capture systems, and observational gait analysis had statistical significance in evaluating the changes in step velocity and step length of lower extremity movement in elderly individuals (*P* < 0.00001), which can be used as a standardized basis for the assessment of motor function in elderly individuals. Subgroup analysis showed that there was significant heterogeneity in the assessment of step velocity [SMD=-0.98, 95%CI(-1.23, -0.72), I^2^ = 91.3%, *P* < 0.00001] and step length [SMD=-1.40, 95%CI(-1.77, -1.02), I^2^ = 86.4%, *P* < 0.00001] in elderly individuals. However, the sensors (I^2^ = 9%, I^2^ = 0%) and 3D motion capture systems (I^2^ = 0%) showed low heterogeneity in terms of step velocity and step length. The sensitivity analysis and publication bias test demonstrated that the results were stable and reliable.

**Conclusion:**

observational gait analysis, motion sensors, 3D motion capture systems, and IMUs, as evaluation means, play a certain role in evaluating the characteristic parameters of step velocity and step length in lower extremity motor function of elderly individuals, which has good accuracy and clinical value in preventing motor injury. However, the high heterogeneity of observational gait analysis and IMUs suggested that different evaluation methods use different calculation formulas and indicators, resulting in the failure to obtain standardized indicators in clinical applications. Thus, multimodal quantitative evaluation should be integrated.

**Supplementary Information:**

The online version contains supplementary material available at 10.1186/s12984-024-01409-7.

## Introduction

According to the World Health Organization (WHO), by 2050, the number of people aged 60 or over will reach 2 billion, and the number of people aged 80 or over will triple to 426 million [1]. As the population ages, diseases and disabilities associated with ageing and behavioral limitations are increasing [2]. Without timely intervention, the risk of sarcopenia, spinal cord injury, and fall injury may increase [3]. The individual and age differences between elderly individual’s affects movement ability and disorder performance differently, such as bradykinesia, myotonia and balance dysfunction, thus increasing the challenge of assessment. At present, the clinical scale is commonly used in the evaluation of lower extremity motor function in elderly individuals. Symptoms, signs and other related indicators were systematically recorded and scored to evaluate the severity of injury and the change of treatment effect. This method is simple to use and does not require expensive professional equipment. For clinicians, information of the patient’s motor function can be obtained initially, which is conducive to the next diagnosis and treatment. However, due to confounding factors, such as physiological, psychological, and behavioral factors of elderly individuals, this scale is not reliably robust, thereby limiting the assessment [4]. How to make an effective and accurate objective quantitative assessment of lower extremity motor function in elderly individuals is still a challenge to overcome for assessing and preventing impairment of the ageing population.

Therefore, the present study selected randomized controlled trials (RCTs) to conduct a meta-analysis of clinical objective detection methods for the assessment of lower extremity motor function in elderly individuals. The detection methods included motion sensors, observational gait analysis, IMUs, 3D motion capture systems, and surface electromyography(sEMG). The purpose of the present study was to summarize the quantitative assessment methods of lower extremity motor function in elderly individuals to provide a reference for clinical application.

## Methods

The present meta-analysis was reported in accordance with the Preferred Reporting Items for Systematic Reviews and Meta-Analyses (PRISMA) statement [5]. The protocol of this systematic review was published in the International Prospective Register of Systematic Reviews (PROSPERO-CRD 42023430185).

### Search strategy

Studies on lower extremity motor function assessment in the elderly were searched from January 2018 to March 2023 in the PubMed, Web of Science, Cochrane Library and EMBASE databases. MeSH terms and text words were included in the search terms. The search strategy was modified for each specific database, with keywords and concepts remaining identical. The main concepts were as follows: elderly, lower extremity, sensors, surface electromyography, inertial measurement units, gait analysis, and motion capture systems. These are currently the most mainstream evaluation methods, with high clinical recognition. A large amount of literature can be retrieved, with high accuracy and reliability. The search strategies for all databases are provided in Table 1.

**Table 1:** Search strategies for all databases

Database	Search strategies	Additional filters
PubMed	(((((((((((Electromyographies[Title/Abstract]) OR (Surface Electromyography[Title/Abstract])) OR (Electromyographies, Surface[Title/Abstract])) OR (Electromyography, Surface[Title/Abstract])) OR (Surface Electromyographies[Title/Abstract])) OR (Electromyogram[Title/Abstract])) OR (Electromyograms[Title/Abstract])) OR (“Electromyography“[Mesh])) OR (((Gait Analyses[Title/Abstract]) OR (Analysis, Gait[Title/Abstract])) OR (“Gait Analysis“[Mesh]))) OR (motion capture system[Title/Abstract])) OR ((inertial sensor[Title/Abstract]) OR (inertial measurement unit[Title/Abstract]))) AND ((((((((((“Lower Extremity“[Mesh]) OR (Extremities, Lower[Title/Abstract])) OR (Lower Extremities[Title/Abstract])) OR (Lower Limb[Title/Abstract])) OR (Limb, Lower[Title/Abstract])) OR (Limbs, Lower[Title/Abstract])) OR (Lower Limbs[Title/Abstract])) OR (Membrum inferius[Title/Abstract])) OR (Extremity, Lower[Title/Abstract])) AND ((Elderly[Title/Abstract]) OR (“Aged“[Mesh])))	Filters: in the last 5 years Sort by: Most Recent
Web of Science	TS=((((((((((((Electromyographies[Title/Abstract]) OR (Surface Electromyography[Title/Abstract])) OR (Electromyographies, Surface[Title/Abstract])) OR (Electromyography, Surface[Title/Abstract])) OR (Surface Electromyographies[Title/Abstract])) OR (Electromyogram[Title/Abstract])) OR (Electromyograms[Title/Abstract])) OR (“Electromyography“[Mesh])) OR (((Gait Analyses[Title/Abstract]) OR (Analysis, Gait[Title/Abstract])) OR (“Gait Analysis“[Mesh]))) OR (motion capture system)) OR ((inertial sensor) OR (inertial measurement unit))) AND ((((((((((“Lower Extremity“[Mesh]) OR (Extremities, Lower[Title/Abstract])) OR (Lower Extremities[Title/Abstract])) OR (Lower Limb[Title/Abstract])) OR (Limb, Lower[Title/Abstract])) OR (Limbs, Lower[Title/Abstract])) OR (Lower Limbs[Title/Abstract])) OR (Membrum inferius[Title/Abstract])) OR (Extremity, Lower[Title/Abstract])) AND ((Elderly[Title/Abstract]) OR (“Aged“[Mesh]))) )	Date of publication: Last 5 years
Cochrane library and Embase	1 MeSH descriptor: [Aged] explode all trees (255,190) 2 (Elderly): ti, ab, kw (57,900) 3 #1 OR #2 (293,731) 4 MeSH descriptor: [Lower Extremity] explode all trees (10,856) 5 (Extremities, Lower): ti, ab, kw OR (Lower Extremities): ti, ab, kw OR (Lower Limb): ti, ab, kw OR (Limb, Lower): ti, ab, kw OR (Limbs, Lower): ti, ab, kw OR (Lower Limbs): ti, ab, kw OR (Membrum inferius): ti, ab, kw OR (Extremity, Lower): ti, ab, kw (23,471) 6 #4 OR #5 (30,797) 7 #3 AND #6 (6841) 8 MeSH descriptor: [Electromyography] explode all trees (4197) 9 (Electromyographies): ti, ab, kw OR (Surface Electromyography): ti, ab, kw OR (Electromyographies, Surface): ti, ab, kw OR (Electromyography, Surface): ti, ab, kw OR (Surface Electromyographies): ti, ab, kw OR (Electromyogram): ti, ab, kw OR (Electromyograms): ti, ab, kw (2910) 10 #8 OR #9 (6043) 11 MeSH descriptor: [Gait Analysis] explode all trees (63) 12 (Gait Analyses): ti, ab, kw OR (Analysis, Gait): ti, ab, kw (4923) 13 #11 OR #12 (4923) 14 (inertial sensor): ti, ab, kw OR (inertial measurement unit): ti, ab, kw (170) 15 (motion capture system): ti, ab, kw (342) 16 #14 OR #15 (507) 17 #10 OR #13 (10,783) 18 #16 OR #17 (11,132) 19 #7 AND #18 (445) 20 #3 AND #18 (2434)	

### Selection criteria

Following Morgan’s PICOS/PECOS program, the inclusion criteria and exclusion criteria were formulated [6]. The inclusion criteria were as follows: (1) study subjects included the elderly aged 60–80 years; and (2) interventions included sEMG, gait analysis, IMUs, 3D motion capture systems, and motion sensors to assess motion function during lower extremity tasks. (3) The multiple comparisons met the following criteria: (a) comparison between the movement disorder group and the healthy group; (b) comparison between the movement characteristic parameters of the elderly before and after the evaluation; and (c) comparison of the lower extremity motor function characteristic results with gold standard clinical scale results. (4) Regarding the outcome indicators: the primary indicator was step velocity, and the secondary indicator was step length. (5) The study was an RCT.

Articles were excluded based on the following criteria: (1) duplicate publications or literature; (2) incomplete research data or test data could not be extracted; (3) review and systematic review; and (4) full text not available.

### Screening, selection process, and data extraction

Two researchers with systematic training independently searched the relevant literature, read the title and abstract of the literature, and conducted preliminary screening according to the inclusion criteria. The full text was then read, and the literature that did not meet the inclusion criteria was screened out. The literature data were extracted, and cross-checks were conducted. Differences were resolved through discussion. The extraction contents included author, year of publication, sample size, age of study subjects, intervention methods, and outcomes.

### Assessment of methodological quality and risk of bias

The risk of bias and methodological quality of the included trials were independently assessed using the Cochrane Risk of Bias (RoB2) assessment tool [7]. RoB2, as proposed by the Cochrane Collaboration, is a widely accepted tool to evaluate the quality of an RCT in the biomedical field. The evaluation items were comprised of the following seven aspects: (1) random sequence generation; (2) allocation concealment; (3) blinding of participants and personnel; (4) blinding of outcome assessment; (5) incomplete outcome data; (6) selective reporting; and (7) other bias. For each indicator, “low risk of bias”, “high risk of bias”, and “unclear” were used to assess bias. If the included studies fully met, partially met, or inconsistently met with the above criteria, the possibility of bias was small, moderate, or high, respectively (quality grades of A, B, or C, respectively).

### Statistical analysis

EndNote X9 was used to deduplicate and filter the literature found in databases. Excel 2010 was used to extract data and basic information. Statistical analysis of the data was performed using RevMan 5.4.1 (The Nordic Cochrane Centre, The Cochrane Collaboration, Copenhagen, Denmark). Continuity variables were extracted from the outcome variables of this study. Measurements were calculated using weighted mean difference (WMD) or standard mean difference (SMD) [8], and the size of the combined effect was analyzed using 95%CI.

A heterogeneity test was first performed. Heterogeneity was evaluated by the I^2^ statistic, which was classified as low, moderate, or high with I^2^ < 25%, 25 − 50%, and > 50%, respectively [9]. If I^2^ < 50% or *P* > 0.05, there was no significant heterogeneity among the included studies, and the fixed effect model was adopted. Otherwise, the random effect model was adopted [10]. *P* < 0.05 was considered statistically significant. With high heterogeneity (I^2^ > 50%), a heterogeneity analysis was performed using subgroups. Stata SE12.0 was used for meta-regression to further explore the sources of heterogeneity. A sensitivity analysis was performed to evaluate the consistency of the results. Begg’s [11] and Egger’s [12] tests were used to evaluate potential publication bias.

## Results

### Study selection

**Fig. 1:**
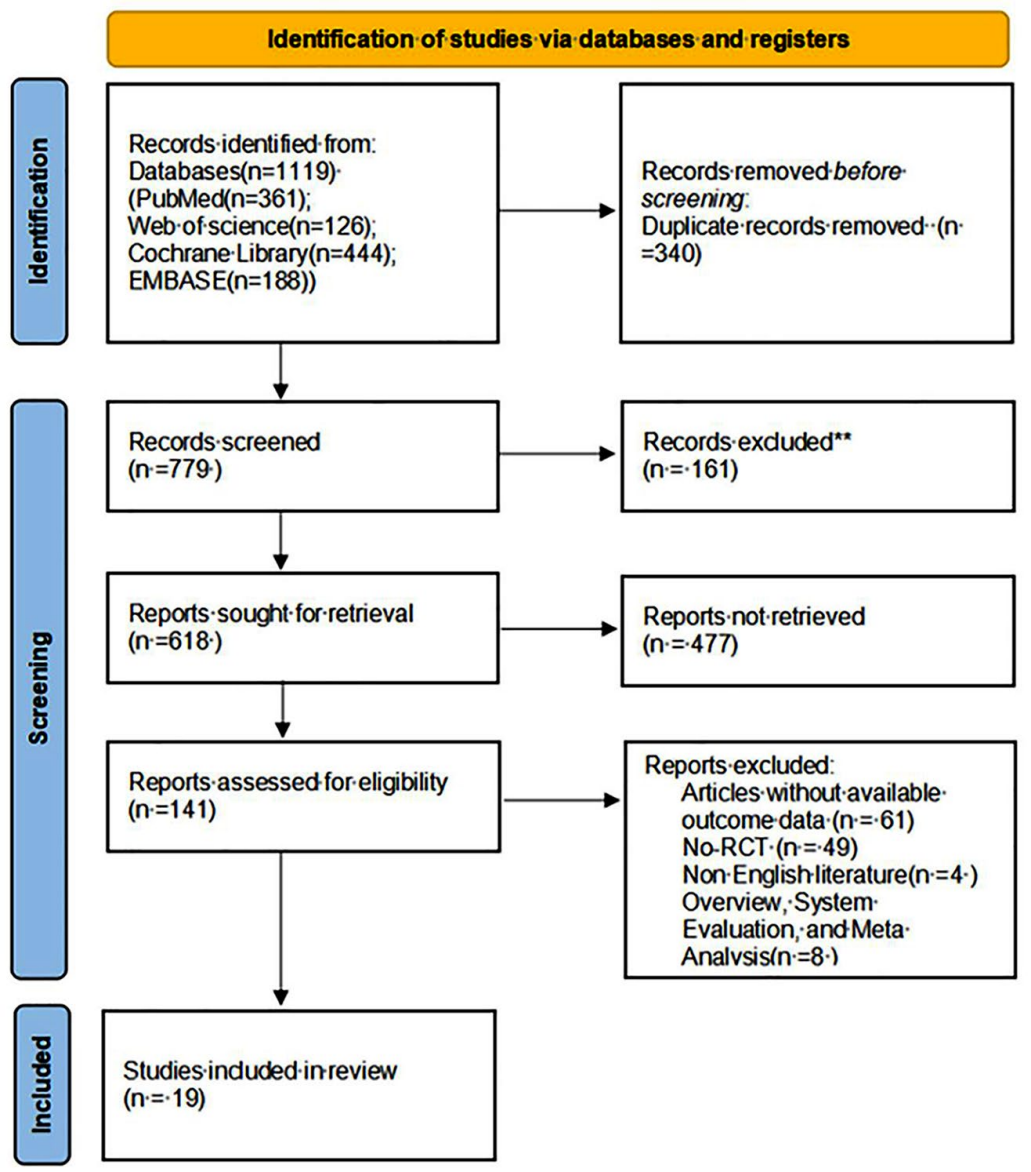
.PRISMA flowchart of the study selection process. RCTs, randomized controlled trials

A total of 1119 studies were initially searched, 340 duplicates were eliminated by Endnote, resulting in 779 papers. After reading the titles and abstracts of the studies, 618 studies were retained. After reading the full text studies that did not meet the inclusion criteria were excluded. Finally, a meta-analysis of 19 studies was conducted (Fig. 1). The sample size was 2626 cases, with 1306 cases in the experimental group and 1320 cases in the control group. The characteristics and outcome measures of each included study are shown in Table 2.

**Table 2:** Characteristics of the randomized controlled studies

References	*N*		Age [mean ± sd or mean (25%, 75% quartiles)]		Type of interventions	Outcome index (m/s, m)
	T	C	T	C		
Beck, Y 2018	101	19	64.5 ± 9.3	77.8 ± 3.9	IMU	step velocity
Ismailidis 2021	22	46	65.9 ± 9.1	66.8 ± 7.4	IMU	step velocity, step length
Lee 2020	74	52	69.6 ± 5.7	71.4 ± 4.9	IMU	step velocity, step length
Noh 2021	290	456	74.2 ± 5.4	72.2 ± 4.9	IMU	step velocity, step length
Qiu 2018	82	142	72.4 ± 4.7	72.0 ± 4.2	IMU	step velocity, step length
Rehman 2020	37	56	70.1 ± 9.3	71.0 ± 7.1	IMU	step velocity
Zago 2018	22	22	69.4 ± 6.1	69.4 ± 6.1	IMU	step velocity, step length
Buongiorno 2019	16	14	74.9 ± 7.6	73.5 ± 6.4	sensor	step velocity, step length
Ismailidis P 2021	24	48	66.1 ± 10.3	66.6 ± 7.2	sensor	step velocity, step length
Morris 2019	18	18	63.4 ± 9.5	63.4 ± 9.5	sensor	step velocity, step length
Gallagher 2019	13	13	68.5 ± 6.3	68.5 ± 6.3	3D motion capture system	step velocity, step length
Maeda 2018	19	30	67.2 ± 9.7	65.1 ± 2.4	3D motion capture system	step velocity, step length
Maier 2019	12	12	61.3 ± 11.4	61.3 ± 11.4	3D motion capture system	step velocity, step length
Wang 2021	44	44	65.7 ± 7.7	65.7 ± 7.7	3D motion capture system	step velocity, step length
Godi 2021	298	84	70.0 ± 7.8	71.5 ± 8.2	Gait analysis	step velocity
Peixoto 2019	33	33	72.7 ± 4.0	72.4 ± 4.0	Gait analysis	step velocity, step length
Perring 2020	15	15	72.3 ± 8.8	73.3 ± 8.8	Gait analysis	step velocity, step length
Rehman 2019	119	184	66.9 ± 10.5	70.0 ± 7.7	Gait analysis	step velocity, step length
Guzik 2020	65	65	61.7 ± 10.8	61.3 ± 11.4	Gait analysis	step length

T, experimental group; C, control group; m/s: meters per second; m: meters

### Risk of bias

**Fig. 2:**
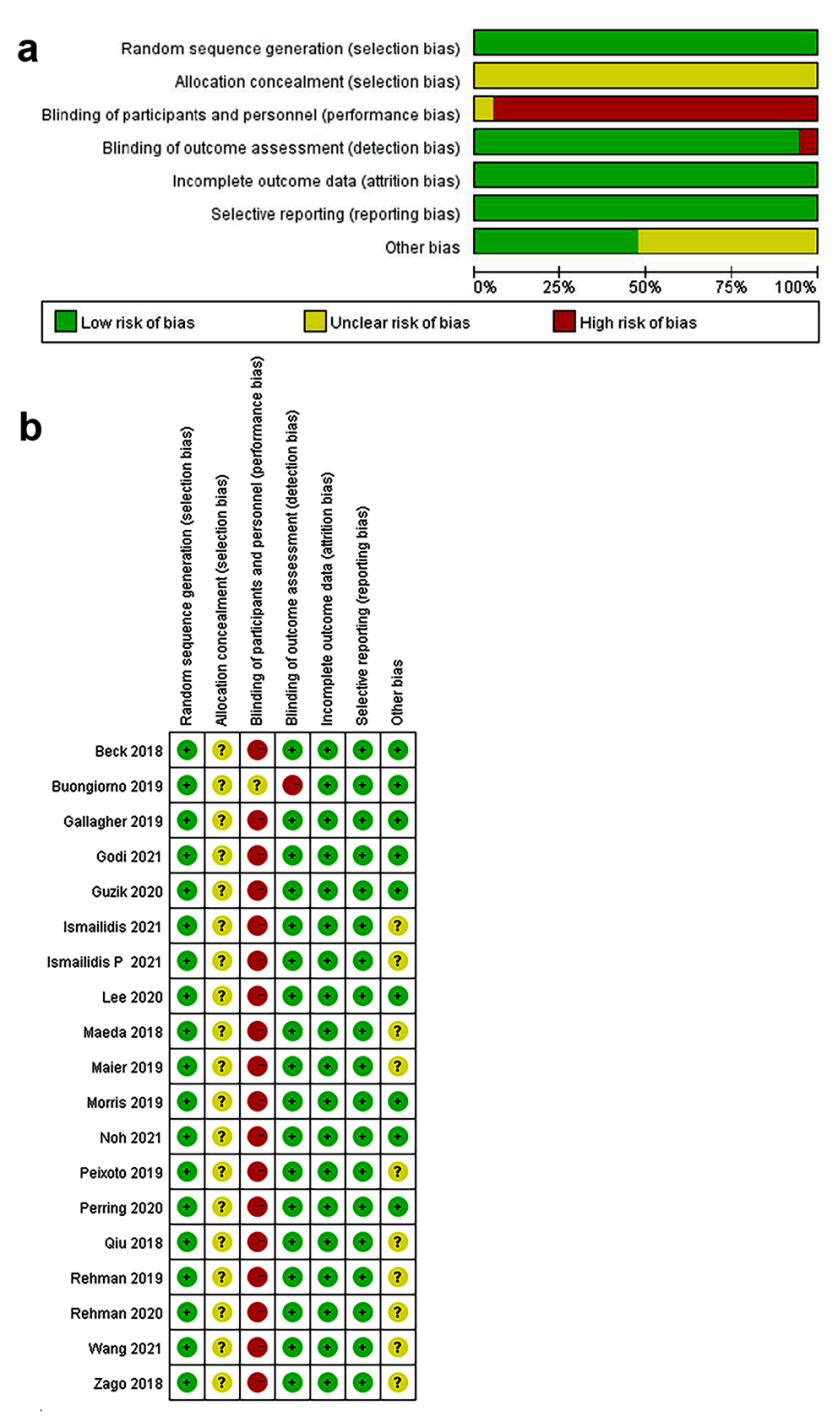
Performance (**a**) and summary plot (**b**) of each type of bias in all studies

The quality of the included literature was evaluated according to the Cochrane Review Manual, and the methodological quality evaluation was graded as B. Regarding random sequence generation, 19 studies described the methods used to generate the sequences in detail and were evaluated as having a low risk of bias. In terms of allocation concealment, none of the 19 studies reported the allocation scheme concealment, which was evaluated as unclear. Regarding blinding, none of the included studies had implemented blinding, and all of the studies were rated as high risk. All studies reported outcomes that were consistent with the study proposal and had a low risk of reporting bias. The included literature reported subjects’ baseline status before intervention, and there were no significant differences between the two groups before intervention. The quality evaluation of the included literature is shown in Fig. 2(a) and (b).

### Meta-analysis

All included studies used step velocity as an outcome measure of lower extremity motor function, and some studies used step length as a secondary measure. The 19 studies involved a total of 2626 elderly individuals, including those with motor dysfunction and healthy elderly individuals. The reliability and validity of the four evaluation methods on the changes in lower extremity motor function characteristic parameters were analyzed.

**Fig. 3:**
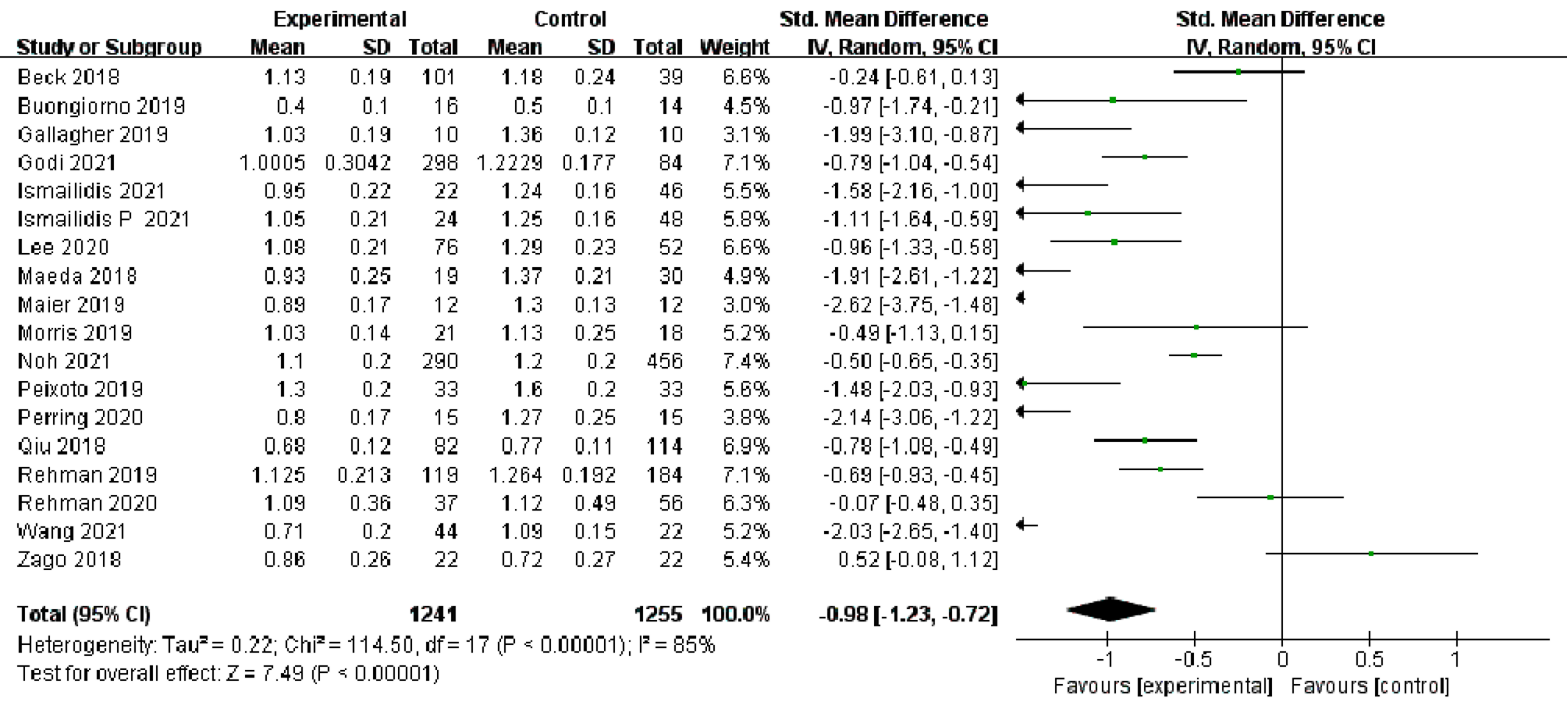
Forest plots of step velocity changes in the elderly by different assessment methods. CI: confidence interval; I2: heterogeneity statistic

**Fig. 4:**
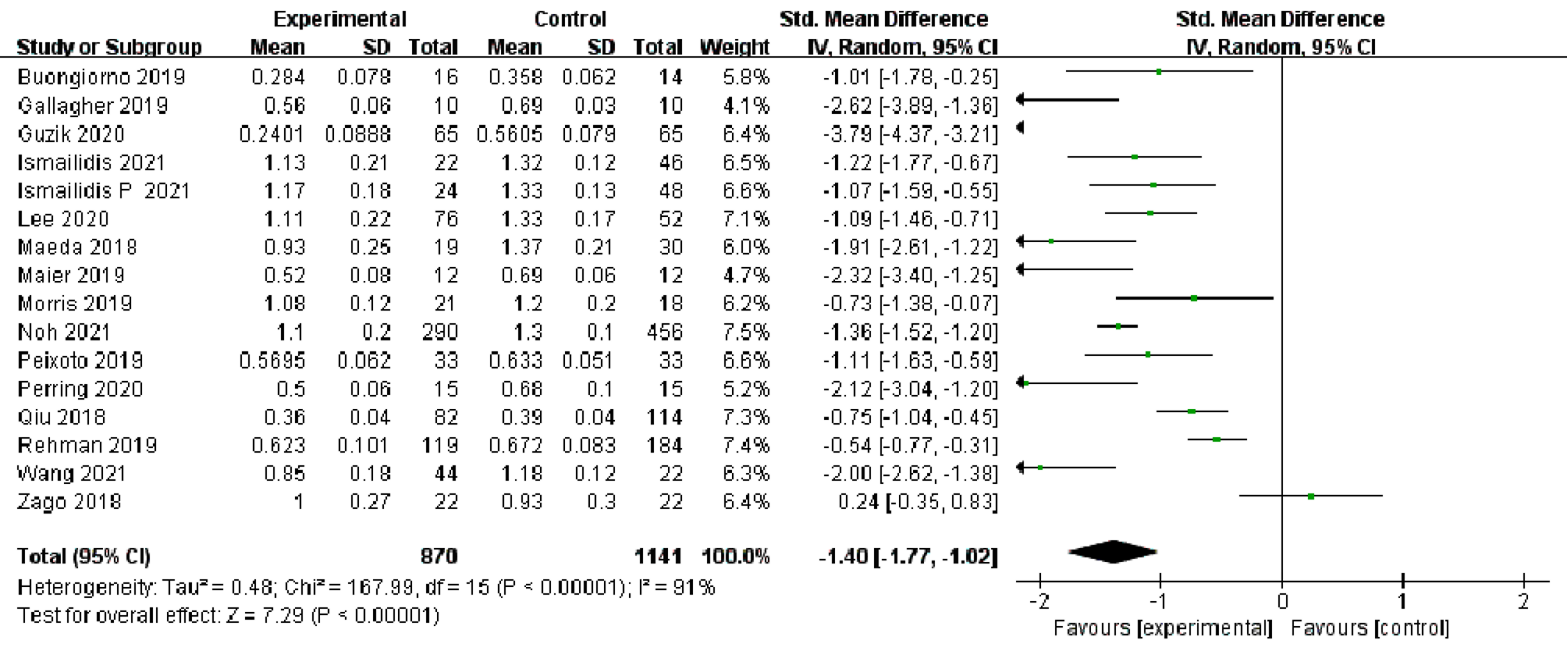
Forest plots of step length changes in the elderly by different assessment methods. CI: confidence interval; I2: heterogeneity statistic

In total, 18 studies [13–30] reported changes in the assessment of step velocity during lower extremity movement in elderly individuals, and 16 studies [13–16, 19–26, 28–31] reported changes in the assessment of step length. Because there was high heterogeneity among different evaluation methods for step velocity (I^2^ = 85%, *P* < 0.00001) and step length (I^2^ = 91%, *P* < 0.00001), a random effects model was selected for analysis. At the same time, there were statistically significant differences in the evaluation of step velocity [SMD=-0.98, 95%CI (-1.23, -0.72), *P* < 0.00001] and step length [SMD=-1.4, 95%CI(-1.77, -1.02), *P* < 0.00001] between the experimental group and the control group, which showed high sensitivity to changes in gait characteristic parameters in elderly individuals (Figs. 3 and 4).

### Subgroup analysis

To further explore the sources of heterogeneity, subgroup analysis of the included literature was performed according to different evaluation methods. The results showed that there was high heterogeneity in the evaluation of step velocity of the elderly by IMUs [SMD=-0.52, 95%CI(-0.86, -0.19), I^2^ = 85%, *P* < 0.00001] and gait analysis [SMD=-1.09, 95%CI(-1.52, -0.67), I^2^ = 79%, *P* < 0.00001]. While sensors [SMD=-0.88, 95%CI(-1.26, -0.51), I^2^ = 9%, *P* < 0.00001] and 3D motion capture systems [SMD=-2.06, 95%CI(-2.46, -1.66), I^2^ = 0%, *P* < 0.00001] showed low heterogeneity. Similarly, in the measurement of step length, the sensors [SMD=-0.95, 95%CI(-1.31, -0.59), I^2^ = 0%, *P* < 0.00001] and 3D motion capture systems [SMD=-2.08, 95%CI(-2.49, -1.68), I^2^ = 0%, *P* < 0.00001] showed low heterogeneity. These results could indicate that sensors and 3D motion capture evaluation methods are highly standardized in clinical use. However, there was high heterogeneity in the IMUs (I^2^ = 88%, *P* = 0.00001) and gait analysis (I^2^ = 97%, *P* = 0.01).

**Fig. 5:**
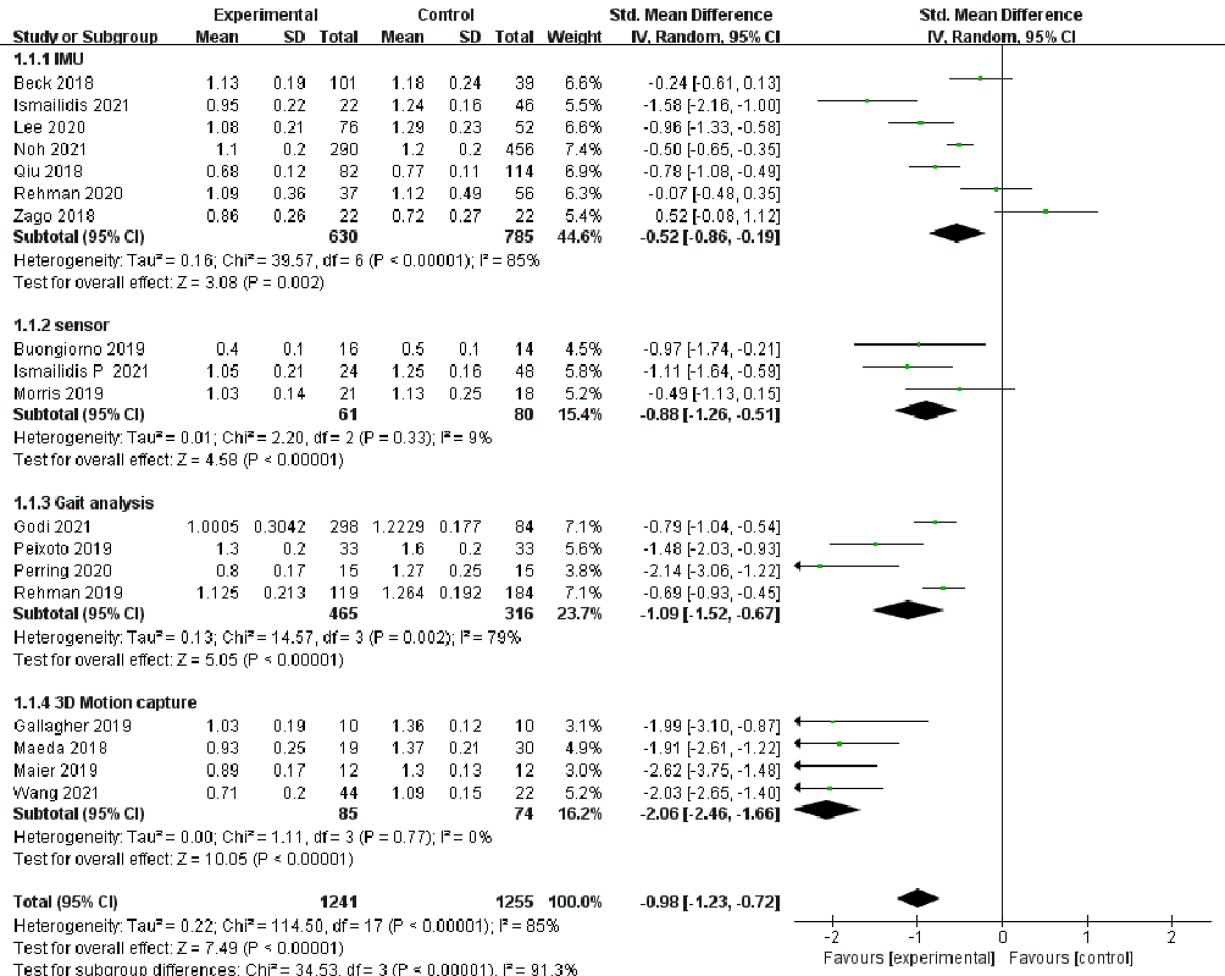
Subgroup analyzes of step velocity changes in the elderly by different assessment methods

**Fig. 6:**
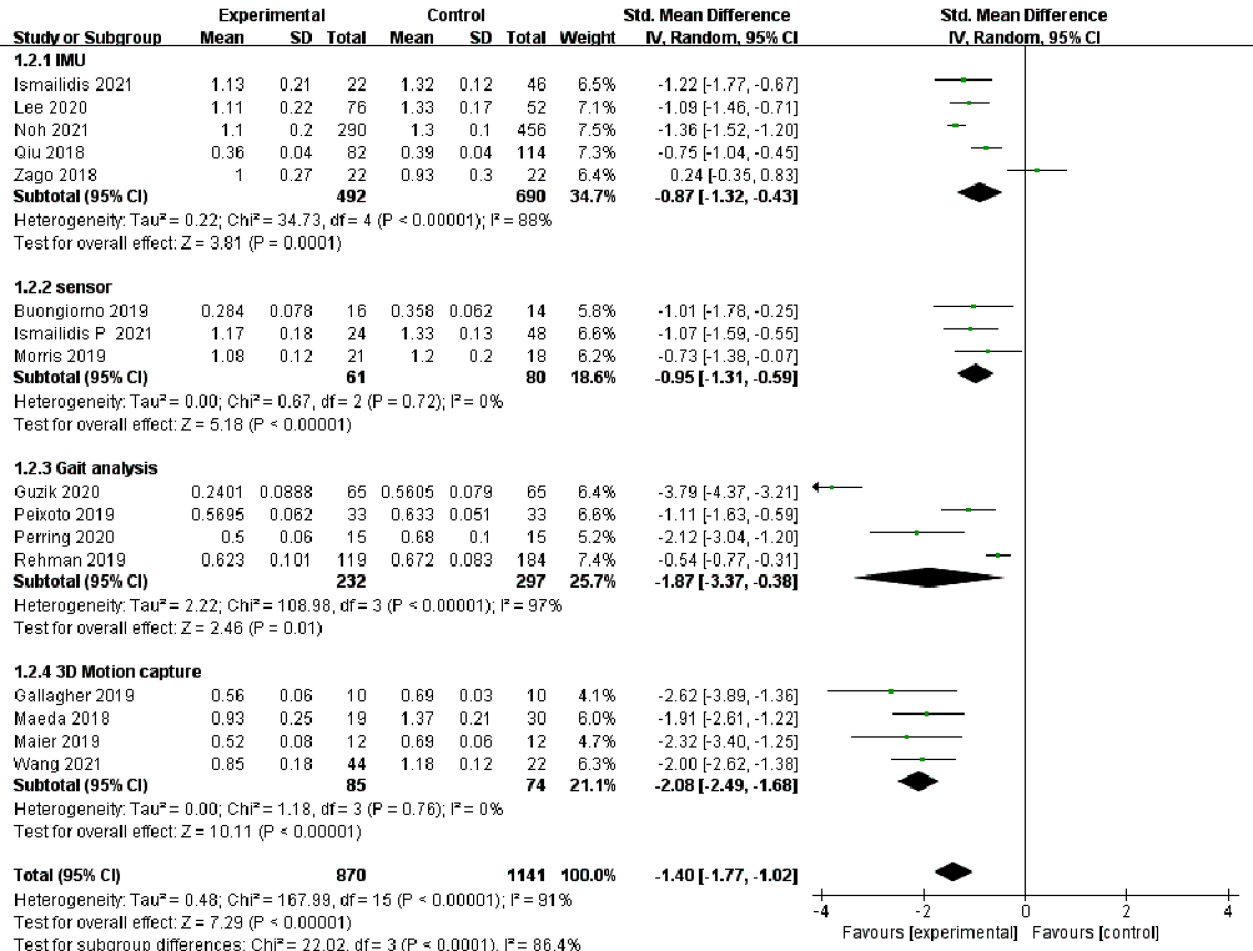
Subgroup analyzes of step length changes in the elderly by different assessment methods

**Fig. 7:**
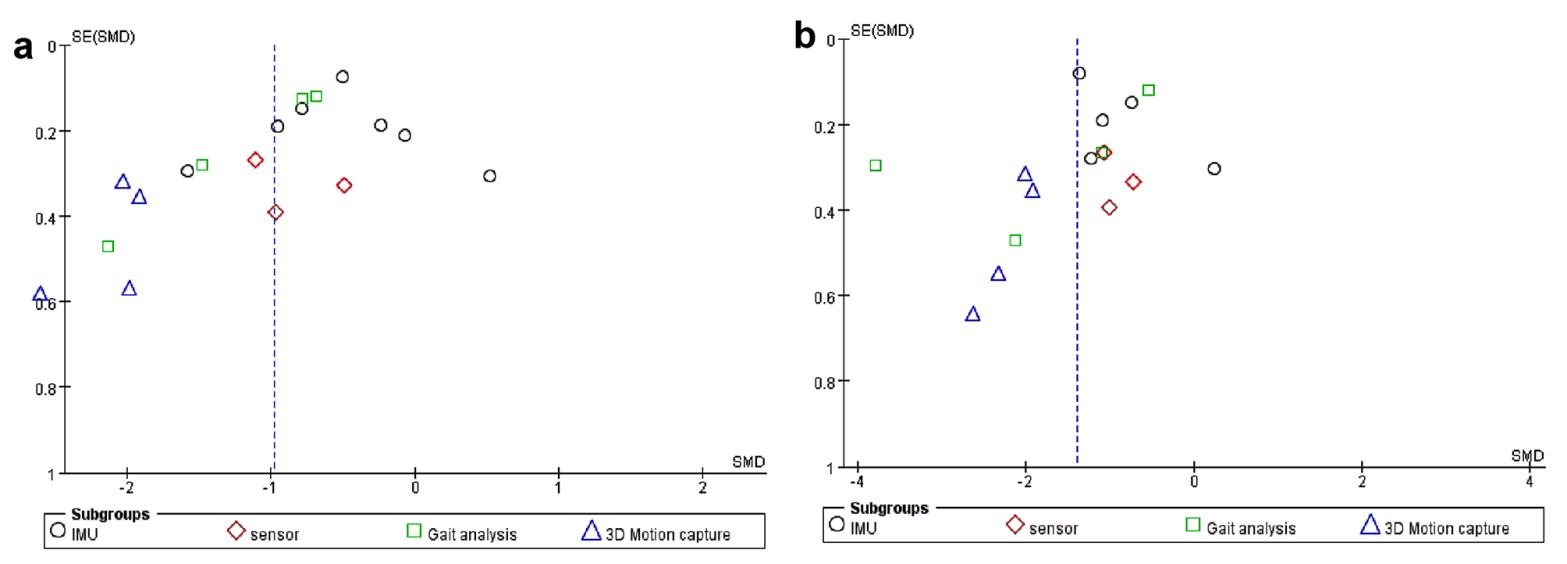
Funnel plot of the changes in characteristic parameters in the elderly by different assessment methods. The left panel (**a**) shows the step velocity result, and the right panel (**b**) shows the step length result

The forest maps showed that there was significant heterogeneity among different research methods for step velocity and length (Figs. 5 and 6). The funnel plot showed that studies were symmetrically distributed on both sides of the combined effect size in the assessment analysis (Fig. 7a and b). This result may be due to the large methodological heterogeneity of the included literature or due to the included studies having a large degree of bias in the aspects of assignment hiding and implement-subject double-blind method. These factors led to large differences in the statistical analysis results.

### Meta Regression

Subgroup analysis showed that there was high heterogeneity in the evaluation of lower extremity characteristic parameters by IMUs and gait analysis. Because fewer than 10 studies were included for gait analysis, the small number of studies may explain this result. Therefore, we did not conduct an in-depth study, but further explored the sources of heterogeneity affecting the evaluation of IMUs and performed a meta-analysis in terms of publication year and country. The results suggested that publication year had a statistically significance effect on heterogeneity (*P* < 0.05), while publication country had no statistically significance effect on heterogeneity (*P* > 0.05). These findings suggested that publication year was one of the source factors of heterogeneity. However, other factors cannot be excluded. The included studies were from 2018, 2020 and 2021, and they showed that the development of IMUs in motor function assessment greatly changed. At the same time, due to the late rise of IMUs and the instability of clinical trials, they are not as widely used in clinical practice as other methods. The details of all studies are provided in Additional file 1.

### Sensitivity analysis

**Fig. 8:**
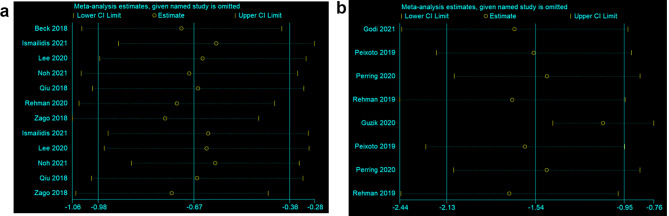
Sensitivity analysis. (**a**) is the IMU and (**b**) is the gait analysis

For the sensitivity analysis, each paper was eliminated one by one for the IMUs and gait analysis with high heterogeneity. The combined effect size was re-estimated and compared to the previous combined effect size excluded. The results showed that the combined effect size had no significant change before and after excluding each article, indicating that the results of this study were stable (Fig. 8a and b).

### Assessment of publication bias

According to the funnel plot results, the studies were symmetrically distributed on both sides of the combined effect size for the evaluation analysis of step velocity and step length. Because some studies deviated from the funnel plot confidence interval, Begg’s and Egger’s tests were necessary to assess publication bias. The results showed that there was significant publication bias between different evaluation methods for step velocity (Egger’s test, t=-2.74, *P* = 0.015; Begg ’s test, *P* = 0.012). Using the random effects model, the heterogeneity test indicated that Q = 119.863 and *P* = 0.000. The combined result of the effect indicators was − 0.998 [95%CI of (-1.259, -0.737)]. Therefore, it was necessary to use the shear compensation method to evaluate the stability of the combined results. After the data of six virtual studies were included, meta-analysis of all studies was performed again. The heterogeneity test indicated that Q = 202.176 and *p* = 0.000. Using the random effects model, the combined result of the effect indicators was − 0.626 [95%CI was (-0.903, -0.348)]. The shear compensation method suggested that the results were statistically significant after the inclusion of six studies. Because there was no reversal, the combined results were robust. The Begg’s and Egger’s tests indicated that step length did not show significant publication bias (Egger’s test, t=-1.13, *P* = 0.277; Begg’s test, *P* = 0.115). The details of all studies are provided in Additional file 2.

## Discussion

The gait data are composed of spatiotemporal parameters, kinematic parameters, dynamic parameters, and myoelectric parameters. The kinematics of elderly individuals can identify abnormal gait [32]. Step velocity is the gait indicator that changes earliest in the elderly and has the highest correlation with daily living ability [33]. What’s more, it is known as the “sixth vital sign,” and its decline is a marker of ageing, various disease states, and early death [34]. Therefore, the present study used step velocity as an index for the lower extremity movement evaluation of the elderly by various evaluation methods. Step length, was also included in the present study to evaluate the motor function of the extremities of elderly individuals.

The RCT results of the 2626 participants included in the present study showed that the IMUs, sensors, 3D motion capture systems, and gait analysis had statistical significance in evaluating the changes in step velocity and step length of lower extremity movement in elderly individuals, which could be used as a standardized basis for the assessment of motor function in elderly individuals. The meta-analysis indicated that sensors and 3D motion capture systems had low heterogeneity, suggesting high reliability in clinical applications. In contrast, IMUs and gait analysis displayed high heterogeneity that was reasonably explained through meta- regression and sensitivity analysis. However, the reliability and consistency of the exercise paradigm of the two assessment methods are still controversial in the assessment of lower extremity exercise in elderly individuals.

Elderly individuals face a enormous risk of motor function impairment [35]. Daily gait assessment can monitor physical conditions that may worsen and prevent future health problems, such as bradykinesia, myotonia, postural instability, tremors and balance dysfunction [36]. The present study demonstrated that although quantitative motor function assessment methods have been applied in clinical practice, there are still human errors, making it difficult to accurately plan follow-up treatment or rehabilitation programs. Different data indicators and methods lead to significant heterogeneity, suggesting that there is a lack of standardized parameters to measure the quality of motor function in the clinic. Thus, multimodal quantitative evaluation needs to be integrated, allowing a better understanding of age-related differences in motor characteristics, which can help to understand gait abnormalities in elderly individuals, and identify therapeutic targets for motor function impairment [37].

### Analysis of highly heterogeneous results from IMUs and gait analysis

The IMU and sEMG discussed in the present study can sense the movement changes or muscle states of the human body, and are widely used. sEMG is a noninvasive and dynamic detection method that accurately reflects muscle activity and function status in real time [38]. However, due to the different muscles involved in different studies and the various considerations of accuracy, the data features included in the selection of normalized analysis vary. Therefore, we did not perform an inductive analysis of the evaluation of lower extremity motor function in elderly individuals. Although the included IMUs showed high heterogeneity, they have good interpretative efficiency for processing changes in gait data [39].

Due to the low cost and high computing power, IMUs have become one of the main means of motor function evaluation. In 2021, using the efficiency and convenience of IMUs, Noh et al. assessed the risk of falls in elderly individuals. Compared to healthy elderly individuals, the results suggested that IMUs are useful in clinical applications. At the same time, integration of IMUs into the Berg balance test, the Up and Go test (TUG), and the six-minute walk test [40] allows assessment of balance outcomes in elderly fall-prone patients [41], as well as helps to prevent falls. Moreover, multidimensional data collection can improve the prediction accuracy of fall risk [42] and provide immediate support for the elderly [43]. All of these results were in agreement with the analysis results of the present study.

The high heterogeneity of IMUs in the present study may be explained by several aspects. First, the calibration problem of IMUs is a major difficulty to overcome. Some authors have proposed corresponding solutions for the major problems of the drift of IMUs estimates and the calibration between IMUs and extremities [44]. However, due to the lack of regulatory standardization of data collection and analysis in the application of IMUs, there has never been a consensus. In addition, due to the lack of standardized data collection and analysis methods, and the high cost and inconvenience of maintenance, the adaptability of IMUs in clinical settings remains limited [45]. Moreover, when assessing the movement state of the extremities, the inherent shortcoming of the need to wear sensors in the fixed position of the body restricts the normal actions of the elderly and prevents them from accurately obtaining movement information in the natural state [46].

Similarly, as another evaluation method with high heterogeneity in the study, the observational gait analysis results may be related to the numerous measurement parameters involved. Due to the fluctuation of an individual’s lower extremity control ability and walking environment changes, gait analysis is manifested as the difference between different individuals, different tests of the same individual, and different gait cycles of the same individual, resulting in inconsistent gait parameters and high variability. Because the ultimate goal of walking is to efficiently move the entire body from one position to another, a quantitative description of the kinematics or dynamics of the whole-body movement during exercise should be the most appropriate method to integrate the local elements of the gait cycle and provide a measure of walking effectiveness. Therefore, it is important to simplify the vast amounts of gait data.

### High evidential reliability of the 3D motion capture system

Because of its objective and quantitative evaluation of human motion function, the 3D motion capture system has potential advantages in accuracy, cost, time, and development difficulty, making it a mainstream human motion measurement method. In different fields, such as medical rehabilitation and motion analysis, the stability, operation efficiency, application flexibility, and system cost reduction of motion capture technology have improved in recent years [47, 48]. 3D motion capture system is divided into mechanical, optical, acoustic and electromagnetic four kinds. Mechanical and optical methods are mainly used to evaluate lower extremity motor function in elderly individuals. The mechanical motion capture system uses the exoskeleton system to record the movement tracks of the human body in real time through sensors such as angle and displacement, so as to accurately present the six degrees of freedom information of the body. The optical motion capture system uses an infrared camera system to identify and process specific light spots by affixing some special “markers” on key parts of the body, such as joints, hips, elbows, wrists, etc., so as to obtain the movement tracks [49]. The four studies included in the present study showed that the 3D motion capture system has gradually demonstrated its superiority in the field of rehabilitation medicine. There is good consistency in the evaluation of kinematic parameters, such as step velocity and step length. At the same time, the low heterogeneity of this method further verifies its clinical feasibility.

### Multimodal data analysis

In the process of collecting literature with step velocity and step length as outcome indicators, we found that the number of literature that could be included was reduced. However, the number of studies that processed these characteristic parameters by means of machine learning and deep learning increased significantly. Multidimensional and multidata systems, such as sEMG and gait analysis, usually generate a large number of high-dimensional measurement data. Because it is not possible to analyse every data point in the clinic, it is necessary to seek appropriate methods to reduce the amount of data analyzed. Machine learning and deep learning have become popular topics in several fields. Studies have indicated that using machine learning and deep learning data processing methods to extract and normalize the extracted record results can provide reliable guidance for clinicians in the diagnosis of motor function in elderly individuals.

Common machine learning algorithms, such as K-nearest neighbour (KNN), support vector machine (SVM), and random forest (RF), have been used to classify elderly fallers with classification accuracies between 69% and 100% [13, 38, 50]. Typical deep learning algorithms include convolutional neural networks (CNNs), recurrent neural networks (RNNs), and deep reinforcement learning (RL). These algorithms have been applied in the assessment of the motor function of elderly extremities by virtue of their strong learning ability, wide coverage, good adaptability, high data-driven upper limit, and good portability. Motion-capture systems and IMUs [51] provide objective contextual information in an automated manner by combining decision trees [50] and deep learning [52]. The trained network has been validated to monitor older adults at increased risk of falls or with any severe gait impairment, with an accuracy of 89.13%. However, deep learning has a high demand for hardware requirements and computing power. Deep learning also requires as much real data as possible to ensure diversity in the dataset, to reduce the impact of interindividual differences in physiological signals, and make the model more robust. Furthermore, the model design is complex, dependent on data, and not highly interpretable. In the case of unbalanced training data, gender and racial discrimination, will occur, and bias easily exists [53–55]. Due to these limitations, deep learning cannot be widely used in the clinic. However, a motion evaluation system combining sensor technology, a three-dimensional motion capture system, gait analysis, and a machine learning algorithm is an inevitable trend in extremity function evaluation, and it is also an inevitable result of promoting rehabilitation medicine in the direction of personalized, accurate, remote and intelligent development.

## Limitations

Although the present study increased the statistical efficiency to a certain extent, there were several limitations. First, due to the small number of included studies and small overall sample size, the efficacy of the meta-analysis results may be insufficient, indicating that additional original studies with large samples are needed. Second, some small sample studies were included in the present meta-analysis, which may affect the credibility of the results. Finally, although we explored possible sources of heterogeneity through subgroup analysis, the sources were not conclusively identified.

Future studies should explore precision rehabilitation as the concept, obtain multisource and multimodal data, and explore the correlation among fusion data, feature selection, and key attribute selection. For the deep learning model, a single outcome index obtained from the fusion of multivariate data, such as time series and spatial series, can be included. The relationship between the multimodal data based on objective detection and the evolution characteristics of the time dimension can be summarized to further analyse the accuracy and effectiveness of various evaluation methods for the detection of motor function in elderly extremities.

## Conclusion

Observational gait analysis, motion sensors, 3D motion capture systems, and IMUs, as evaluation means, play a certain role in evaluating the characteristic parameters of step velocity and step length in lower extremity motor function of elderly individuals, which has good accuracy and clinical value in preventing motor injury. However, the high heterogeneity of gait analysis and IMUs suggested that different evaluation methods use different calculation formulas and indicators, resulting in the failure to obtain standardized indicators in clinical applications. Thus, multimodal quantitative evaluation needs to be integrated. A deep learning algorithm may improve high heterogeneity by extracting and normalizing feature vectors, but the high requirements for data collection limit its clinical application. It is necessary to further explore the application limitations of each evaluation method and to refine the interrelationship and time-dimensional evolution characteristics of multimodal data based on objective detection. Through comprehensive and homogenized datasets, the accuracy and effectiveness of various evaluation methods for the detection of motor function in the elderly extremities can be improved.

### Electronic supplementary material

Below is the link to the electronic supplementary material.

Supplementary Material 1

Supplementary Material 2

## Data Availability

All data generated or analyzed during this study are included in this published article and its Additional files.

## Abbreviations

WHO: World Health Organization
IMU: Inertial measurement unit
sEMG: Surface electromyography
RCTs: Randomized controlled trials
3D motion capture system: Three-dimensional motion capture system
PRISMA: Preferred Reporting Items for Systematic Reviews and Meta-Analyses
WMD: Weighted mean difference
SMD: Standard mean difference
KNN: K-nearest neighbour
SVM: Support vector machine
RF: Random forest
CNN: Convolutional neural networks
RNN: Recurrent neural networks
RL: Deep reinforcement learning
