# Supplementary material for: Meta-analysis of the quantitative assessment of lower extremity motor function in elderly individuals based on objective detection
Source: J Neuroeng Rehabil. 2024 Jun 26;21:111. doi: 10.1186/s12984-024-01409-7 (PMC11202321; doi:10.1186/s12984-024-01409-7)
Supplement: Supplementary file 1 — Supplementary Material 1 [file 12984_2024_1409_MOESM1_ESM.docx]

Table 1 Results of meta-regression analysis of heterogeneity factors affecting IMU

| Research Features | regression coefficient | 95%CI | t value | P |
| --- | --- | --- | --- | --- |
| Year | -0.074 | [-0.134, -0.014] | -2.79 | 0.021* |
| country | 0.044 | [-0.012, 0.101] | 1.79 | 0.107 |

CI: confidence interval; * indicates P<0.05, which was considered to indicate statistical significance.
