# Supplementary material for: Meta-analysis of the quantitative assessment of lower extremity motor function in elderly individuals based on objective detection
Source: J Neuroeng Rehabil. 2024 Jun 26;21:111. doi: 10.1186/s12984-024-01409-7 (PMC11202321; doi:10.1186/s12984-024-01409-7)
Supplement: Supplementary file 2 — Supplementary Material 2 [file 12984_2024_1409_MOESM2_ESM.docx]

**Publication bias**

**Part A is the Begg's and Egger's test result of step velocity.**

metabias _ES _seES

Note: default data input format (theta, se_theta) assumed.

Tests for Publication Bias

Begg's Test

adj. Kendall's Score (P-Q) = -67

Std. Dev. of Score = 26.40

Number of Studies = 18

z = -2.54

Pr > |z| = 0.011

z = 2.50 (continuity corrected)

Pr > |z| = 0.012 (continuity corrected)

Egger's test

| Std_Eff | Coef. | Std. Err. | t | P>\|t\| | [95% Conf. | Interval] |
| --- | --- | --- | --- | --- | --- | --- |
| slope | -0.2732904 | 0.1900632 | -1.44 | 0.170 | -0.6762063 | -0.1296256 |
| bias | -2.7947 | 1.0199 | -2.74 | 0.015 | -4.95679 | -0.6326095 |

**Part B shows the result of cutting and compensating method of step velocity.**

|  | Pooled | 95% CI | | Asymptotic | | No. of |
| --- | --- | --- | --- | --- | --- | --- |
| Method | Est | Lower | Upper | z_value | p_value | studies |
| Fixed | -0.718 | -0.804 | -0.631 | -16.336 | 0.000 | 18 |
| Random | -0.998 | -1.259 | -0.737 | -7.493 | 0.000 |  |

Meta-analysis

Test for heterogeneity: Q= 119.863 on 17 degrees of freedom (p= 0.000)

Moment-based estimate of between studies variance = 0.236

Trimming estimator: Linear

Meta-analysis type: Fixed-effects model

| iteration | estimate | Tn | # to trim | diff |
| --- | --- | --- | --- | --- |
| 1 | -0.718 | 128 | 5 | 171 |
| 2 | -0.636 | 135 | 6 | 14 |
| 3 | -0.613 | 136 | 6 | 2 |
| 4 | -0.613 | 136 | 6 | 0 |

Filled

Meta-analysis

|  | Pooled | 95% CI | | Asymptotic | | No. of |
| --- | --- | --- | --- | --- | --- | --- |
| Method | Est | Lower | Upper | z_value | p_value | studies |
| Fixed | -0.613 | -0.696 | -0.530 | -14.481 | 0.000 | 24 |
| Random | -0.626 | -0.903 | -0.348 | -4.413 | 0.000 |  |

Test for heterogeneity: Q= 202.716 on 23 degrees of freedom (p= 0.000)

Moment-based estimate of between studies variance = 0.374

**Part C is the Begg's and Egger's test result of step length.**

metabias _ES _seES

Note: default data input format (theta, se_theta) assumed.

Tests for Publication Bias

Begg's Test

adj. Kendall's Score (P-Q) = -36

Std. Dev. of Score = 22.21

Number of Studies = 16

z = -1.62

Pr > |z| = 0.105

z = 1.58 (continuity corrected)

Pr > |z| = 0.115 (continuity corrected)

Egger's test

| Std_Eff | Coef. | Std. Err. | t | P>\|t\| | [95% Conf. | Interval] |
| --- | --- | --- | --- | --- | --- | --- |
| slope | -0.8642682 | 0.3169599 | -2.73 | 0.016 | -1.54408 | -0.1844569 |
| bias | -1.777562 | 1.572581 | -1.13 | 0.277 | -5.150412 | 1.595288 |
